# Staphylococcus pseudintermedius Surface Protein L (SpsL) Is Required for Abscess Formation in a Murine Model of Cutaneous Infection

**DOI:** 10.1128/IAI.00631-18

**Published:** 2018-10-25

**Authors:** Amy C. Richards, Marie O'Shea, Philippa M. Beard, Mariya I. Goncheva, Stephen W. Tuffs, J. Ross Fitzgerald, Andreas Lengeling

**Affiliations:** a The Roslin Institute, Royal (Dick) School of Veterinary Sciences, University of Edinburgh, Edinburgh, United Kingdom; University of Illinois at Chicago

**Keywords:** Staphylococcus pseudintermedius, abscess, cellulitis, skin infection

## Abstract

Staphylococcus pseudintermedius is the leading cause of pyoderma in dogs and is often associated with recurrent skin infections that require prolonged antibiotic therapy. High levels of antibiotic use have led to multidrug resistance, including the emergence of epidemic methicillin-resistant clones. Our understanding of the pathogenesis of S. pseudintermedius skin infection is very limited, and the identification of the key host-pathogen interactions underpinning infection could lead to the design of novel therapeutic or vaccine-based approaches for controlling disease. Here, we employ a novel murine cutaneous-infection model of S. pseudintermedius and investigate the role of the two cell wall-associated proteins (SpsD and SpsL) in skin disease pathogenesis. Experimental infection with wild-type S. pseudintermedius strain ED99 or a gene-deletion derivative deficient in expression of SpsD led to a focal accumulation of neutrophils and necrotic debris in the dermis and deeper tissues of the skin characteristic of a classical cutaneous abscess. In contrast, mice infected with mutants deficient in SpsL or both SpsD and SpsL developed larger cutaneous lesions with distinct histopathological features of regionally extensive cellulitis rather than focal abscessation. Furthermore, comparison of the bacterial loads in S. pseudintermedius-induced cutaneous lesions revealed a significantly increased burden of bacteria in the mice infected with SpsL-deficient mutants. These findings reveal a key role for SpsL in murine skin abscess formation and highlight a novel function for a bacterial surface protein in determining the clinical outcome and pathology of infection caused by a major canine pathogen.

## Abstract (precis)

Staphylococcus pseudintermedius is the leading cause of pyoderma in dogs and is often associated with recurrent skin infections that require prolonged antibiotic therapy. High levels of antibiotic use have led to multidrug resistance, including the emergence of epidemic methicillin-resistant clones.

## INTRODUCTION

Staphylococcus pseudintermedius, a coagulase-positive species, is a natural commensal of the skin and mucosal membranes of dogs, with healthy carriage rates reported to range from 46% to 92% (1). S. pseudintermedius is capable of causing a range of opportunistic infections, including urinary tract, ear, wound, and surgery-related infections (1). The most clinically important consequence of S. pseudintermedius infection is canine pyoderma, with approximately 10% of dogs affected worldwide (2). Canine pyoderma is an umbrella term used to describe a range of clinical manifestations, most commonly superficial bacterial folliculitis and atopic dermatitis, which are often treated with antibiotics alongside topical creams and shampoos containing antimicrobial agents, such as chlorhexidine (3, 4). The repeated use of antibiotics in patients with recurrent pyoderma is linked to the development of antibiotic resistance and the rapid global spread of methicillin-resistant S. pseudintermedius (MRSP), with some strains developing resistance to all the antimicrobials commonly used in veterinary medicine (5–8). The high prevalence of multidrug-resistant S. pseudintermedius is a major concern for the continued treatment of canine pyoderma.

Our understanding of the pathogenesis of S. pseudintermedius infection and the key bacterial factors involved is very limited. The exfoliative toxins ExpA and ExpB cause intraepidermal clefts in the skin by directly cleaving canine desmoglein 1, and both intradermal and subcutaneous injection of either exfoliative toxin in dogs leads to the development of clinical manifestations of pyoderma, including crusting and erythema (9–11). Bacterial adhesins are also thought to be important, as the S. pseudintermedius clinical isolate ED99 demonstrates increased adherence to pyoderma-associated canine corneocytes compared with healthy corneocytes (12). Of the 18 cell wall-associated (CWA) proteins encoded in the genome sequence of S. pseudintermedius clinical isolate ED99, two have been demonstrated to mediate binding to host proteins (13). S. pseudintermedius surface proteins D and L (SpsD and SpsL) mediate binding to the host extracellular matrix proteins fibrinogen and fibronectin and the cytoskeletal protein cytokeratin-10 when expressed by the heterologous host Lactococcus lactis (13). Recombinant versions of the SpsD N-terminal A domain interfere with fibrin clot formation and platelet aggregation *in vitro*, and both SpsD and SpsL are sufficient for the invasion of S. pseudintermedius into canine progenitor epidermal keratinocytes in a fibronectin-dependent manner *in vitro* (14, 15). To date, the roles of S. pseudintermedius putative virulence factors have not been examined during experimental infection.

Skin infection models have been employed previously to analyze the roles of CWA proteins of Staphylococcus aureus by subcutaneous injection of wild-type and gene deletion strains (16–18). In these studies, mice developed focal skin abscesses and dermonecrosis within 24 h that subsequently resolved spontaneously after 14 days in the absence of a systemic response (16). These skin lesions can be evaluated using histopathology or homogenized to determine the number of viable bacteria present. Here, we developed the first murine infection model of S. pseudintermedius and assessed the roles of SpsD and SpsL in this murine model using single- and double-deletion strains of *spsD* and *spsL* in the S. pseudintermedius clinical isolate ED99, originally isolated in the United Kingdom from a dog affected by canine pyoderma (12). We discovered that the mice infected with the wild-type and *spsD*-deficient strains developed classical abscessation near the inoculation site. In contrast, mice inoculated with *spsL*- or *spsL spsD*-deficient strains developed an alternative clinical pathology described as cellulitis. These findings demonstrate that a bacterial CWA protein determines the clinical outcome of infection in a novel murine cutaneous model.

## RESULTS

### SpsD and SpsL of S. pseudintermedius ED99 adhere to murine fibronectin, fibrinogen, and cytokeratin-10.

**FIG 1:**
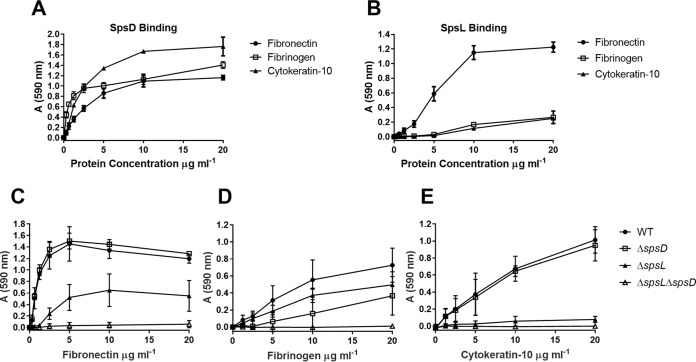
SpsD and SpsL promote adherence to murine fibronectin, fibrinogen, and cytokeratin-10. (A and B) Bacterial solid-phase adherence assays of stationary-phase L. lactis expressing SpsD (A) and L. lactis expressing SpsL (B) to murine fibronectin, fibrinogen, and cytokeratin-10. (C to E) Bacterial solid-phase adherence assays of ED99 wild type (WT), ED99 Δ*spsD*, ED99 Δ*spsL*, and ED99 Δ*spsL* Δ*spsD* to murine fibronectin (C), murine fibrinogen (D), and murine cytokeratin-10 (E) at early exponential growth phase (OD_600_ = 0.2). Each data point represents the mean value from three independent experiments; the error bars represent standard deviations (SD).

Previous work demonstrated that S. pseudintermedius SpsD and SpsL can mediate binding to the extracellular matrix proteins fibronectin and fibrinogen and the cytoskeletal protein cytokeratin-10 when expressed on the cell surface of a heterologous host, L. lactis (2). To determine if murine skin was a suitable model for the analysis of SpsD and SpsL, we examined their capacity to mediate binding to the murine proteins fibronectin, fibrinogen, and cytokeratin-10. The expression of SpsD and SpsL on the surface of L. lactis mediated binding to murine fibronectin, fibrinogen, and cytokeratin-10, with SpsD demonstrating stronger binding to murine cytokeratin-10 and fibrinogen than SpsL (Fig. 1A and B). This suggests that the roles of SpsD and SpsL in the pathogenesis of S. pseudintermedius canine pyoderma can be examined in a murine infection model.

In order to examine the importance of SpsD and SpsL in mediating binding of S. pseudintermedius ED99 to the same murine ligands, we performed solid-phase bacterial-adherence assays with the wild type and *spsL* and *spsD* single- or double-deletion mutants (15). Expression analysis demonstrated that SpsD is expressed on the cell surface at early exponential phase (optical density at 600 nm [OD_600_], 0.2), with SpsL expressed on the cell surface throughout the exponential phase (data not shown). Accordingly, the binding potentials of CWA SpsD and SpsL were investigated by solid-phase adherence assays at early exponential growth phase. As expected, S. pseudintermedius ED99 demonstrated adherence to murine fibronectin, murine fibrinogen, and murine cytokeratin-10 (Fig. 1C to E). Equivalent murine fibronectin binding to the wild type was observed for ED99 Δ*spsD*, demonstrating that SpsL promotes binding to fibronectin (Fig. 1C). However, ED99 Δ*spsL* retained a reduced adherence to fibronectin, demonstrating that SpsD is sufficient for fibronectin binding (Fig. 1C). Adherence to murine fibrinogen was reduced in comparison to the other ligands with both ED99 Δ*spsD* and ED99 Δ*spsL* exhibiting reduced binding, suggesting that both SpsL and SpsD are capable of mediating adherence to murine fibrinogen (Fig. 1D). Surprisingly, ED99 Δ*spsL* exhibited poor binding to cytokeratin-10, suggesting that SpsL is the main mediator of cytokeratin-10 binding (Fig. 1E). Of note, ED99 Δ*spsL* Δ*spsD* demonstrated complete ablation of binding to all three ligands, confirming that SpsD and SpsL are the only CWA proteins of S. pseudintermedius ED99 promoting adherence to murine fibronectin, fibrinogen, and cytokeratin-10 under these assay conditions (Fig. 1A to C).

### Development of a murine cutaneous-infection model of S. pseudintermedius ED99.

**FIG 2:**
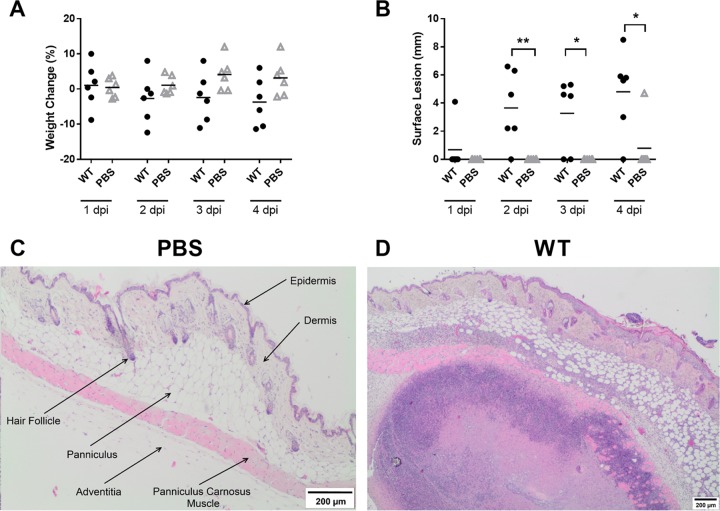
S. pseudintermedius ED99-infected mice develop skin abscesses. (A and B) Monitoring data are shown as percent change in body weight (A) and surface lesion length (B) for ED99 WT-infected mice and PBS control mice. Each data point represents an individual mouse. Mean scores (horizontal bars) were analyzed by two-sample unpaired *t* test. *, *P* ≤ 0.05; **, *P* ≤ 0.01. (C and D) Representative images of H&E-stained skin sections from a PBS control mouse and a WT-infected mouse at 4 dpi. A hair follicle and all 5 layers of the skin are labeled in the PBS control image. The WT image contains a large, well-circumscribed abscess in the deeper layers of the skin. The abscess is composed of a necrotic center surrounded by a concentric ring of neutrophils and cellular debris accompanied by suppurative dermatitis, panniculitis, myositis, and myofiber degeneration.

Initially, pilot experiments were performed to develop the first murine skin infection model of S. pseudintermedius. Female BALB/c mice received an injection of either a 100-μl volume of phosphate-buffered saline (PBS) or 1 × 10^7^ CFU of S. pseudintermedius ED99 (in 100 μl PBS) as a bolus into the subcutaneous tissue of the dorsal midline, just caudal to the interscapular region. The mice were then monitored for 4 days postinfection (dpi) to determine the severity of the disease and to follow the development of skin lesions. The wild-type-infected mice exhibited a trend toward greater weight loss than the control mice, but it was not significant, suggesting that a systemic infection did not occur (Fig. 2A), and there were no differences in the sizes of excised spleens (see Fig. S1A in the supplemental material). Cutaneous lesions in the wild-type-infected mice became visible on the back or flank of the mouse at 2 dpi and became more prominent by 4 dpi (Fig. 2B). The gross pathology was typified by soft cutaneous lesions of various shapes up to 9 mm along the longest axis, unattached to the underlying tissue and with a well-defined, reddened margin. On the cut surface there were one or 2 circumscribed, round, soft to liquid areas (pus) approximately 1 to 2 mm in diameter within the dermis and subcutis underlying the lesion.

Subsets of mice were euthanized at 1, 2, and 4 dpi, and the dorsal cutaneous tissue was excised, fixed in 10% formal saline, and processed using standard procedures into tissue sections, which were then stained with hematoxylin and eosin (H&E) and examined by a veterinary pathologist (P.M.B.). No significant pathology was identified in the tissue from the control mice (Fig. 2C). Wild-type-infected mice developed classic skin abscesses characterized by a focal accumulation of neutrophils (some degenerate), cellular debris, and bacteria (Fig. 2D), with no appreciable differences in size or disease severity over time (see Fig. S1B and C in the supplemental material). This pilot study demonstrated that S. pseudintermedius-infected mice develop classic cutaneous abscesses similar to those clinically described in dogs affected by S. pseudintermedius pyoderma (3).

### Infection with S. pseudintermedius lacking SpsD and SpsL cell wall-associated proteins results in a distinct skin infection pathology.

**FIG 3:**
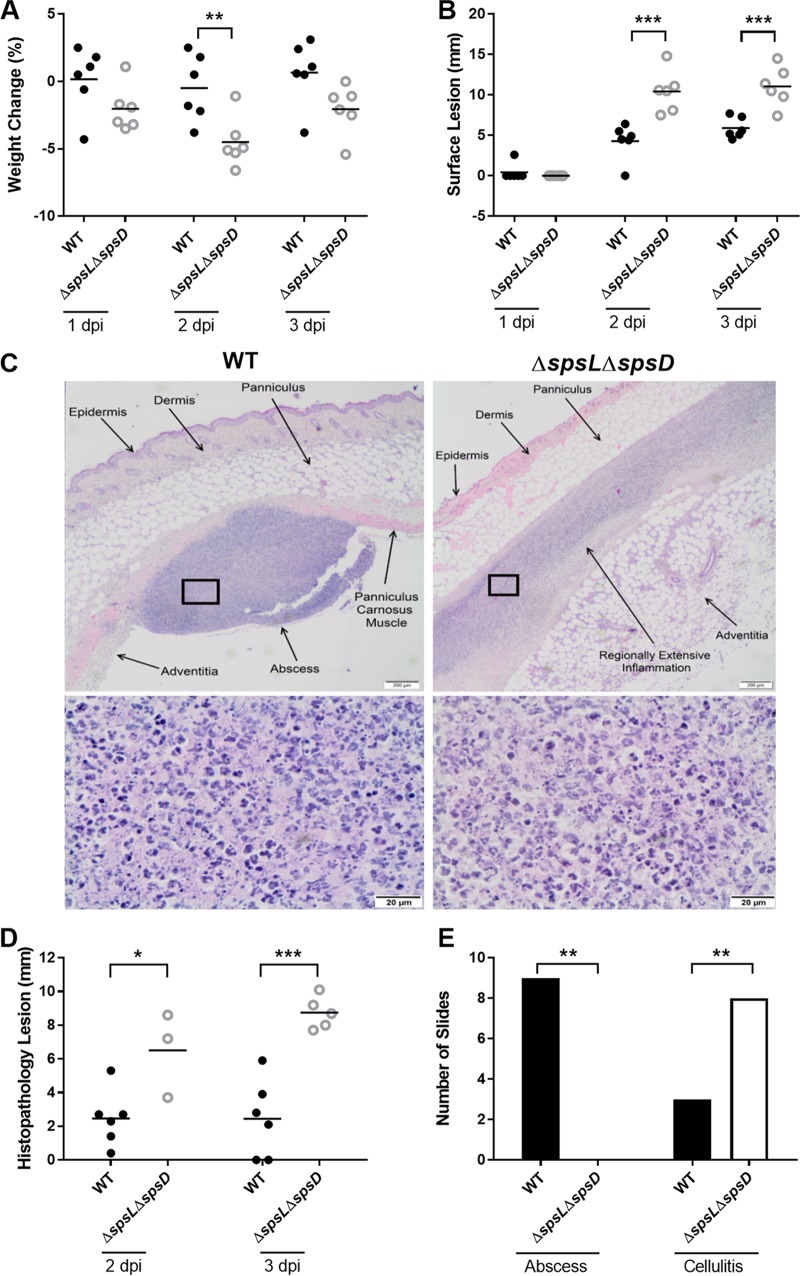
Infection with S. pseudintermedius ED99 deficient in SpsD and SpsL leads to the development of large surface lesions and cellulitis. (A and B) Monitoring data are shown as percent change in body weight (A) and surface lesion length (B) for WT- and ED99 Δ*spsL* Δ*spsD*-infected mice. Each data point represents an individual mouse, and the data are representative of a single replicate of experiments performed twice. Mean scores (horizontal bars) were analyzed by two-sample unpaired *t* test. (C) (Top) Representative images of H&E-stained skin sections of WT-infected or ED99 Δ*spsL* Δ*spsD*-infected mice at 3 dpi. The WT-infected mice displayed a focal abscess. The ED99 Δ*spsL* Δ*spsD*-infected mice displayed a horizontally oriented inflammation throughout the panniculus carnosus muscle layer akin to cellulitis. (Bottom) Magnification (×100) of the boxed areas showing that, in all experimental groups, the regions of inflammation are composed predominantly of degenerate neutrophils admixed with cellular debris. (D) Histopathology lesion lengths. (E) Number of mice demonstrating each pathology type. No abscesses were documented in the ED99 Δ*spsL* Δ*spsD*-infected mice, and the inflammation present was more longitudinally extended than in WT-infected mice. Fisher's exact analysis demonstrated differences in infection outcome between WT- and ED99 Δ*spsL* Δ*spsD*-infected mice. Significant results are represented. *, *P* ≤ 0.05; **, *P* ≤ 0.01; ***, *P* ≤ 0.001.

The murine S. pseudintermedius skin infection model was used to determine the role of bacterial CWA proteins in the pathogenesis of cutaneous infection. Female BALB/c mice were injected subcutaneously, as described previously, with either S. pseudintermedius wild type or the derivative ED99 Δ*spsL* Δ*spsD* deletion mutant and monitored for 3 dpi. The mice infected with the ED99 Δ*spsL* Δ*spsD* strain lost more body weight than the mice infected with the wild-type strain at all three time points (*P* ≤ 0.05 at 2 dpi) (Fig. 3A). Gross examination of the site of inoculation revealed that the wild-type-infected mice developed raised, soft cutaneous lesions with a reddened margin similar to those in the pilot experiments described above. In comparison, ED99 Δ*spsL* Δ*spsD*-infected mice developed flattened, longitudinally extended cutaneous lesions that covered a larger area (see Fig. S2 in the supplemental material). Measurement of the cutaneous lesions along the longest axis confirmed that ED99 Δ*spsL* Δ*spsD*-infected mice developed significantly longer lesions than wild-type-infected mice at both 2 and 3 dpi (*P* ≤ 0.001) (Fig. 3B). Histopathological examination of the inoculated area in mice infected with the wild-type strain revealed the presence of focal, well-circumscribed abscesses consistent with those observed in the pilot study (Fig. 3C). However, focal-abscess formation was not observed in the ED99 Δ*spsL* Δ*spsD*-infected mice, which instead revealed cellulitis characterized by poorly delineated, regionally extensive areas of suppurative inflammation accompanied by abundant necrotic debris, particularly in the deeper cutaneous layers (Fig. 3C). The inflammatory infiltrate extended laterally between tissue planes. Both distinct pathology types, abscessation and cellulitis, contained neutrophils as the predominant inflammatory cell (Fig. 3C). No differences in spleen length or infection severity between infection groups were recorded, with wide within-group variation observed (see Fig. S3A and B in the supplemental material). The categorization of cellulitis was correlated with larger histopathological lesions in ED99 Δ*spsL* Δ*spsD*-infected mice than in wild-type-infected mice at both 2 (*P* ≤ 0.05) and 3 (*P* ≤ 0.0001) dpi (Fig. 3D). Categorizing the histopathological changes into abscessation or cellulitis confirmed decreased abscess formation in ED99 Δ*spsL* Δ*spsD*-infected mice (*P* ≤ 0.005) (Fig. 3E). These data suggest that formation of a focal abscess after cutaneous inoculation in mice is dependent on expression of one or more CWA proteins.

### SpsL is required for the development of skin abscesses in a murine infection model.

**FIG 4:**
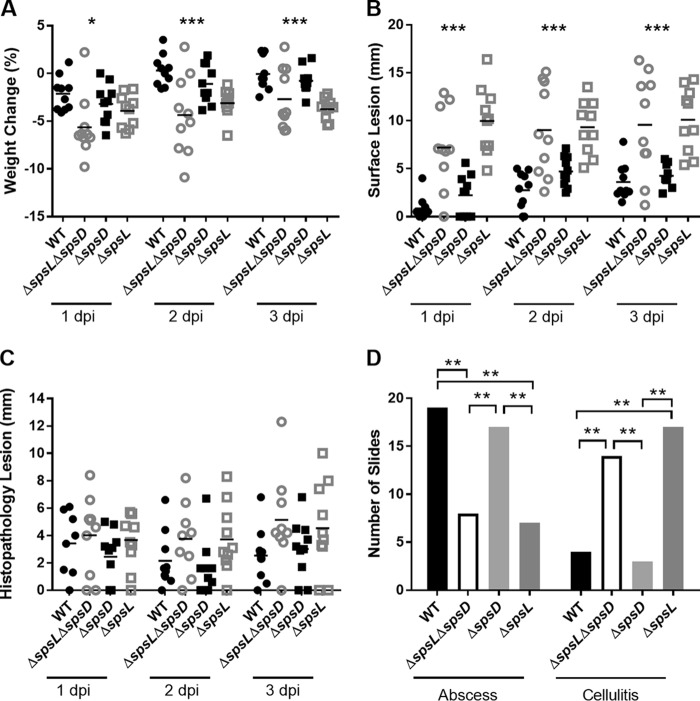
SpsL is required for S. pseudintermedius abscess formation. (A and B) Monitoring data are shown as percent change in body weight (A) and surface lesion length (B) for WT-, ED99 Δ*spsL* Δ*spsD*-, ED99 Δs*psD*-, and ED99 Δ*spsL*-infected mice. Each data point represents an individual mouse, and the data are representative of a single replicate of experiments performed twice. Mean scores (horizontal bars) were analyzed by one-way ANOVA. (C) Histopathology lesion length analyzed by Kruskal-Wallis test. (D) Number of mice demonstrating each pathology type. Fisher's exact analysis demonstrated differences in infection outcome for each experimental group compared to every other experimental group. Significant results are represented. *, *P* ≤ 0.05; **, *P* ≤ 0.01; ***, *P* ≤ 0.001.

In order to investigate the relative role of SpsD and SpsL, single-gene deletion mutants, ED99 Δ*spsD* and ED99 Δ*spsL*, were employed. Ten female BALB/c mice were inoculated with either the wild-type, ED99 Δ*spsD*, ED99 Δ*spsL*, or ED99 Δ*spsL* Δ*spsD* strain of S. pseudintermedius as described above; monitored; and euthanized at 1, 2, or 3 dpi. Measurement of body weight and cutaneous-lesion length demonstrated that ED99 Δ*spsD*-infected mice displayed characteristics more similar to those of the wild-type-infected mice, while both the ED99 Δ*spsL*- and ED99 Δ*spsL* Δ*spsD*-infected mice displayed decreased body weight (Fig. 4A) and increased lesion length (*P* ≤ 0.001) (Fig. 4B) in comparison to wild-type-infected mice. Although upon histopathological examination no differences in histopathology lesion length were identified (Fig. 4C), clear differences in the type of histopathology present were observed between experimental groups (Fig. 4D). The wild-type- and ED99 Δ*spsD*-infected mice were more likely to develop abscesses, and the ED99 Δ*spsL*- and ED99 Δ*spsL* Δ*spsD*-infected mice were more likely to develop cellulitis (*P* ≤ 0.01) (Fig. 4D).

**FIG 5:**
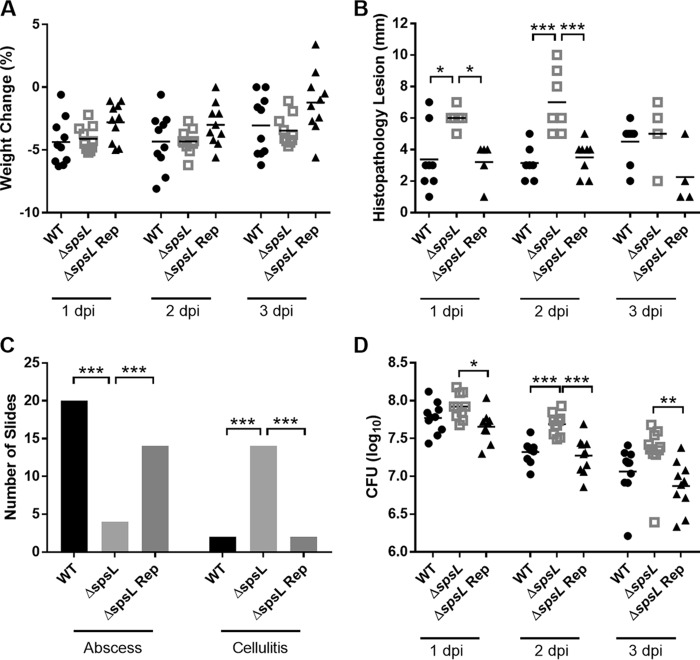
Reintroduction of *spsL* to S. pseudintermedius restores the abscess phenotype. (A) Percent change in body weight. Mean scores (horizontal bars) were analyzed by one-way ANOVA. (B) Histopathology lesion lengths. Mean scores were analyzed by Kruskal-Wallis test. (C) Number of mice demonstrating each pathology type. Fisher's exact analysis demonstrated differences in infection outcome for each experimental group compared to every other experimental group. (D) Log CFU per lesion. Mean values were analyzed by one-way ANOVA with Tukey's multiple-comparison analysis. Each data point represents an individual WT-, ED99 Δ*spsL*-, or ED99 Δ*spsL* Rep-infected mouse. Significant results are represented. *, *P* ≤ 0.05; **, *P* ≤ 0.01; ***, *P* ≤ 0.001.

To confirm the role of SpsL in abscess formation and to fulfil molecular Koch's postulates (19), the *spsL* gene was reintroduced into the ED99 Δ*spsL* strain by allele replacement as described in Materials and Methods. The ED99 Δ*spsL* repaired (Rep) strain demonstrated restored SpsL surface expression and bacterial adherence to canine fibrinogen, allowing analysis in the murine model alongside the wild-type and ED99 Δ*spsL* strains using the same experimental setup described above (see Fig. S4 in the supplemental material). Gross examination identified no differences in the body weights of mice from all the experimental groups (Fig. 5A). However, there were raised, soft cutaneous lesions with a reddened margin present at the inoculation site of the ED99 Δ*spsL* Rep-infected mice, similar to the wild-type-infected mice, with the ED99 Δ*spsL*-infected mice demonstrating flattened cutaneous lesions that were significantly longer at 1 (*P* ≤ 0.05) and 2 (*P* ≤ 0.001) dpi (Fig. 5B). Histopathological examination confirmed that ED99 Δ*spsL* Rep-infected mice were more likely to develop abscesses than ED99 Δ*spsL*-infected mice (*P* ≤ 0.001) (Fig. 5C). These data indicate that SpsL is the first S. pseudintermedius virulence factor to be described and that SpsL plays a key role in determining the pathology of S. pseudintermedius infection in this murine cutaneous model.

### Cellulitis is linked with increased bacterial load.

**FIG 6:**
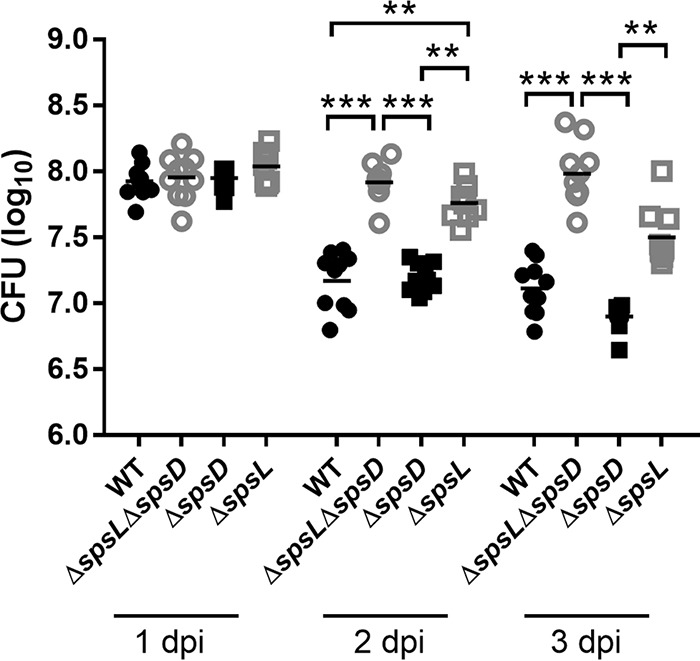
The cellulitis pathology induced by S. pseudintermedius deficient in SpsL is associated with increased bacterial numbers. The numbers of CFU were analyzed after homogenization of the excised and trimmed skin lesion. The log CFU per lesion data are plotted, with each data point representing an individual WT-, ED99 Δ*spsL* Δ*spsD*-, ED99 Δs*psD*-, or ED99 Δ*spsL*-infected mouse. Mean values (horizontal bars) were analyzed by one-way ANOVA with Tukey's multiple-comparison analysis. **, *P* ≤ 0.01; ***, *P* ≤ 0.001. Mice infected with bacterial strains lacking the *spsL* gene had increased bacterial burdens at 2 and 3 dpi.

In order to determine the number of viable bacteria associated with the two observed pathology types, the above-described experiment involving 4 experimental groups was repeated. At 1, 2, and 3 dpi, the mice were euthanized, and the cutaneous lesion on the dorsal midline was excised, homogenized, and cultured to determine the number of live bacteria present in the tissue (total number of CFU per lesion). The same gross observations were made relating to larger lesions in ED99 Δ*spsL*- and ED99 Δ*spsL* Δ*spsD*-infected mice in comparison to wild-type- and ED99 Δ*spsD*-infected mice (data not shown). In addition, increased bacterial loads were identified in ED99 Δ*spsL*- and ED99 Δ*spsL* Δ*spsD*-infected mice in comparison to wild-type- and ED99 Δ*spsD*-infected mice at 2 and 3 dpi (*P* ≤ 0.001) (Fig. 6). Importantly, mice infected with a strain with a restored *spsL* gene, ED99 Δ*spsL* Rep, had fewer bacteria present in the cutaneous lesion than ED99 Δ*spsL*-infected mice at 1 (*P* ≤ 0.05), 2 (*P* ≤ 0.001), and 3 (*P* ≤ 0.01) dpi and with numbers similar to those in wild-type-infected mice (Fig. 5D). These data reveal an association between pathology and bacterial burden that is dependent on SpsL.

## DISCUSSION

The global dissemination of multidrug-resistant MRSP strains is making it increasingly difficult to treat canine pyoderma. There are also increasing reports of human-related infections caused by S. pseudintermedius and colonization of veterinary staff (20–22). Similar to dogs, skin and soft tissue infections, including abscess formation, are the most common forms of disease caused by S. pseudintermedius in humans (20, 22). However, some more invasive infections have also been reported, including bloodstream infection and cellulitis (20, 23). If much-needed novel therapeutics are to be developed to combat MRSP, then a greater understanding of the critical host-pathogen interactions leading to the development of canine pyoderma and human skin and soft tissue infections is required. Here, we have established the first murine model of S. pseudintermedius skin infection. Subcutaneous injection of S. pseudintermedius ED99 led to the development of focal skin abscesses (Fig. 2B) similar to those observed after subcutaneous injection of S. aureus (24). We found that, while SpsD is dispensable, SpsL is required for the development of classic S. pseudintermedius skin abscesses in the murine model.

A common feature of S. aureus cutaneous-infection models is the development of dermonecrosis, depending on the inoculum dose and bacterial strain used (16, 25). S. aureus dermonecrosis is dependent on toxins, such as the alpha-toxin and the α-type phenol-soluble modulins, with mice infected with strains deficient in the *hla* or *psmα* gene unable to develop dermonecrosis (26–28). No dermonecrosis was evident in this study, but necrosis was identified that typically involved the epidermis but that was also variably associated with underlying cutaneous layers. This necrosis of the epidermis was present in all the experimental groups and was not associated with the development of a particular type of pathology. This demonstrates that the development of cellulitis is not linked to changes in the level of epidermal necrosis and that the dermonecrosis discussed in the S. aureus literature is not akin to the cellulitis phenotype described here.

Clinically, abscess formation and cellulitis are distinct phenotypes that can present simultaneously and that can be both acute and chronic (24). Abscesses encase the bacteria in a confined location, while cellulitis is more likely to affect a larger area of the skin with diffuse infiltrations of neutrophils (29). The development of cellulitis in dogs is rare, but cases of S. pseudintermedius-mediated cellulitis have been reported, with lymphatic damage linked to bacteremia and toxic shock or necrotizing fasciitis (30–32). There is little understanding of how either an abscess or cellulitis pathology develops, but it has been thought that the host immune response has a pivotal role in driving the development of each pathology (29). The work presented here suggests that bacterial surface proteins can determine the pathological outcome of infection.

For S. aureus-mediated skin abscesses or microabscesses formed after bloodstream infection, a number of bacterial proteins have been implicated in the development of the abscesses (33). The secreted coagulase and von Willebrand binding protein (vWbp) are required for cutaneous abscess formation in both mice and rabbits (34, 35). In addition, a sortase mutant lacking expression of CWA proteins, as well as strains with deletions of particular genes, such as *clfA*, has been employed to demonstrate the role of CWA proteins in the development of skin abscesses (16). However, in contrast to what we observed with SpsL in the current study, loss of expression did not lead to the development of a cellulitis pathology. These data suggest that SpsL exhibits a function not previously observed among other staphylococcal surface proteins.

The most abundant extracellular matrix protein in healthy skin is collagen. However, when skin is damaged, such as during subcutaneous inoculation, the initiation of the coagulation cascade leads to platelet activation, resulting in high levels of fibrinogen and fibronectin at the inoculation site (36, 37). We postulate that SpsL promotes abscess formation through the development of a fibrinogen or fibronectin shield on the bacterial surface that could initiate bacterial aggregation. The importance of bacterial aggregation for the initial stages of skin abscess development has already been reported for S. aureus, with bacterial aggregates linked to increased bacterial load and bacterial dissemination (38). In S. aureus, this aggregation is coagulase dependent, with a lack of aggregation leading to a decreased bacterial burden (38). In contrast, SpsL-dependent abscess formation results in a decreased bacterial burden at 2 and 3 dpi in comparison to the alternative cellulitis pathology (Fig. 6). The widespread conservation of the *spsL* gene in S. pseudintermedius strains from across the world suggests that S. pseudintermedius could favor the development of subcutaneous abscesses rather than cellulitis (7, 13, 39, 40). Experiments performed over a longer period could determine if S. pseudintermedius is able to persist and avoid clearance in the SpsL-dependent abscesses in comparison to the cellulitis pathology. Additionally, bacterial aggregation could be beneficial to S. pseudintermedius in other types of infection, and particularly in the initial stages of biofilm formation, which may promote colonization and persistence on indwelling devices or atopic skin (41, 42).

Ideally, the role of SpsL in abscess formation and the mechanism involved could be further examined using a canine infection model. A canine superficial pyoderma model has been developed allowing the application of bacteria onto artificially created skin abrasions (43). This model produces classical clinical signs of pyoderma, including the development of pustules and dermatitis that was self-limiting (43). It would be interesting to investigate the roles of both SpsD and SpsL in this model, as their roles in cell invasion *in vivo* could also be established.

In conclusion, by developing the first S. pseudintermedius murine model, we have been able to examine the roles of the 2 CWA proteins of S. pseudintermedius, SpsD and SpsL, in the development of cutaneous infections. We have found that SpsL promotes the development of abscesses, and this is the first description of a bacterial CWA protein influencing the type of cutaneous pathology developed during experimental infection. As so little is known about the immune mechanisms responsible for the development of either an abscess or cellulitis pathology, the model developed here could be useful for future investigations into the molecular basis of these clinical manifestations. More work on the *in vivo* functions of virulence factors of S. pseudintermedius is needed if we aim to develop novel therapeutics to combat the increasingly multidrug-resistant clinical strains of S. pseudintermedius.

## MATERIALS AND METHODS

### Ethics statement.

All murine experiments were carried out under the authority of a United Kingdom Home Office Project License (PPL 70/08663) within the terms and conditions of the strict regulations of the United Kingdom Home Office Animals (Scientific Procedures) Act 1986 and the code of practice for the housing and care of animals supplied for scientific purposes. In all experiments, female BALB/cANCrl (Charles River) (referred to here as BALB/c) mice aged between 10 and 12 weeks were used. All the mice were housed under specific-pathogen-free (SPF) conditions at the Biological Research Facility (BRF) for rodents at the Roslin Institute according to hygiene recommendations of the Federation of European Laboratory Animal Science Associations (FELASA) guidelines (44, 45). Mice were randomly assigned to individually ventilated home cages after arrival and acclimatized for 1 to 2 weeks in the facility before being used in infection challenge studies. Throughout maintenance and infection challenge, the animals had ad libitum access to food and water. The mice were maintained on a standard diet (Teklad Global 18% Protein Rodent Diet), at an average room temperature of 21°C and a 12-h/12-h light/dark cycle. All study protocols were reviewed by the Roslin Institute (University of Edinburgh) animal services, consisting of the named veterinary surgeon (NVS), the rodent facility director, and a senior research statistician, prior to each experiment. The animals were monitored twice daily to ensure that no animal exceeded agreed euthanasia criteria, including a 20% loss of body weight or moderate signs of general illness. Only mild symptoms of generalized illness were noted, with slight decreases in weight, some discharge from the eyes, and starring of the fur. No animal required premature euthanasia, and all the animals were humanely euthanized at the end of experimentation by schedule 1 asphyxiation using carbon dioxide (VetTech Solutions). The euthanized animals were then subjected to cervical dislocation to ensure euthanasia.

### Solid-phase adherence assays.

Solid-phase adherence assays were performed as described previously (13). Briefly, 96-well MaxiSorp plates (Nunc) were coated overnight with murine fibrinogen (Abcam), murine fibronectin (Abcam), or recombinant murine cytokeratin-10 C terminus (residues 294 to 570, purified as described previously [46]) at 4°C in PBS. The wells were blocked with 100 μl 8% (wt/vol) nonfat dry milk (Fluka Analytical) for 2 h at 37°C. L. lactis strains, characterized previously (13), were cultured overnight in M17 broth (Oxoid), washed in PBS, and diluted to an OD_600_ of 1.0 in PBS. S. pseudintermedius strains, characterized previously (15), were cultured in brain heart infusion broth (Oxoid) to an OD_600_ of 0.6, washed in PBS, and diluted to an OD_600_ of 1.0 in PBS. Bacteria were applied to the wells in triplicate and incubated for 2 h at 30°C for L. lactis or for 2 h at 37°C for S. pseudintermedius. Bound bacteria were fixed with 100 μl 25% (vol/vol) formaldehyde (Sigma-Aldrich) for 30 min and then stained with 50 μl 0.5% (wt/vol) crystal violet (Sigma-Aldrich) for 3 min. Before analysis, 5% (vol/vol) acetic acid (BDH Lab Supplies) was applied, and the plate was read using a Synergy HT plate reader (BioTek) at a wavelength of 590 nm.

### Murine skin infection model.

The bacterial inoculum was produced by culturing S. pseudintermedius strains to an OD_600_ of 0.6 in brain heart infusion broth (Oxoid); 10 ml of the culture was washed and then diluted in PBS to the relevant OD_600_ to provide 10^7^ CFU per 100 μl. The size of the inoculum used for infection challenge was confirmed by CFU plating for each experiment.

The dorsal midlines of isoflurane-anesthetized BALB/c mice were shaved using electric clippers 24 h pre-inoculum administration. Bacteria were injected into the subcutaneous tissue of the dorsal midline just caudal to the intrascapular region of the BALB/c mouse in a 100-μl volume using a 26-gauge needle and a 5-mm needle guard to ensure standard injection depths. The mice were caged with 6 or fewer mice per cage, with at least 6 mice per experimental group. All animals were monitored for weight and length of the observed lesion along the longest axis using calipers, noting the presence or absence of a lump, as well as identifying any clinical signs of illness. The longest axis of the lesion was deemed the most appropriate measure, as the lesions developed were highly irregular in shape, meaning that measurement of the lesion width was very subjective. Mice were humanely euthanized at 1, 2, 3, or 4 dpi. Spleens and skin lesions were immediately excised and immersed in 10% formal buffered saline (Fisher Scientific) for histopathological analysis. All experiments were repeated at least twice, giving the same results.

Skin lesions were trimmed, and histopathology slides were produced by the Pathology Department of the Royal Dick School of Veterinary Studies, University of Edinburgh, using routine methods. The histology slides were stained with H&E, staining nuclei blue and proteinaceous material pink. The histopathology slides were analyzed blind for lesion length along the longest axis using a magnifying glass and calipers, as well as for disease severity, and categorized depending on the type of pathology present (abscess/cellulitis). This analysis was independently performed by two persons before unblinding. All histopathology slides included for analysis contained the following 5 layers of skin: epidermis, dermis, panniculus, panniculus carnosus muscle, and adventitia. The histopathology slides were removed from the analysis if only superficial layers of the skin were present on the slide.

For CFU determination, after excision of the skin lesion, the area was sliced into equal sections using a scalpel. These skin sections (containing the whole skin lesion) were suspended in 1 ml PBS in lysing matrix D tubes containing 1.4-mm ceramic spheres (MP Bio). After calculating the weight of the skin lesion, homogenization of the skin samples was performed by pulsing the samples at 4.0 m/s twice for 20 s per pulse with a 1-minute break between pulses. Triplicate serial dilutions were produced per homogenate and plated for CFU enumeration.

### Histopathology severity scoring.

The overall severity of pathology present on the histopathology slide was scored using the following system: grade 0, minimal pathological changes; grade 1, mild inflammatory changes with a mild increase in the number of neutrophils and lymphocytes present in the panniculus and deeper connective tissue layers; grade 2, moderate pathology with formation of either a focal, well-defined abscess in the skin accompanied by inflammation in surrounding tissues or laterally oriented cellulitis with a poorly defined accumulation of neutrophils and cell debris extending between the tissue planes; grade 3, marked pathology displaying more extensive pathology, with the abscess or cellulitis effacing a larger area of the section and overlying epidermal ulceration often noted; and grade 4, severe pathology with large-abscess or cellulitis formation, epidermal ulceration, and disruption of normal tissue architecture.

### Generation of the ED99 Δ*spsL* Rep strain.

Sequence ligase-independent cloning was used to clone the full-length *spsL* gene, along with 500-bp flanking regions, into the temperature-sensitive allele replacement vector pIMAY as previously described (47, 48). A synonymous mutation was introduced into the N-terminal region of the *spsL* gene to allow identification in comparison to the wild type (primer sequences are given in Table S1 in the supplemental material). The pIMAY plasmid containing the mutated *spsL* gene was electrotransformed into the S. pseudintermedius ED99 Δ*spsL* background at 28°C with selection on 10-μg/ml chloramphenicol. Growth at 37°C selected for plasmid integration before plasmid excision at 28°C. The antisense *secY* mechanism was found to be nonfunctional in S. pseudintermedius, as previously described (15). Generation of the ED99 Δ*spsL* Rep strain was confirmed by PCR and Sanger sequencing, as well as Western blot analysis. CWA protein profiles of strains cultured to an OD_600_ of 0.6 in brain heart infusion broth, produced as described previously (15), were probed with 1 μg/ml anti-SpsL N2N3 IgY and 0.5 μg/ml F(ab′)_2_ rabbit anti-chicken horseradish peroxidase (HRP)-conjugated IgG (Bethyl Laboratories).

### Statistical analysis.

Prism 6 (GraphPad) was employed to present data, with statistical analysis performed using Minitab 16. The data from each experiment and each time point were analyzed separately, with data between time points not pooled except to analyze the pathology type data. The Anderson-Darling test was used to assess data normality and equal variance. Data transformation was performed if required and analyzed using either a 2-sample unpaired *t* test or one-way analysis of variance (ANOVA), with multiple comparisons performed when appropriate. If the data could not be successfully transformed, nonparametric analysis was performed, including Kruskal-Wallis or Mann-Whitney U test analysis. For analysis of severity grading, ordinal logistic regression was performed, with pathology type data analyzed in pairs using Fisher's exact analysis.

## Supplementary Material

Supplemental file 1
