# Supplementary material for: Staphylococcus pseudintermedius Surface Protein L (SpsL) Is Required for Abscess Formation in a Murine Model of Cutaneous Infection
Source: Infect Immun. 2018 Oct 25;86(11):e00631-18. doi: 10.1128/IAI.00631-18 (PMC6204706; doi:10.1128/IAI.00631-18)
Supplement: Supplemental file 1 [file zii999092586s1.pdf]

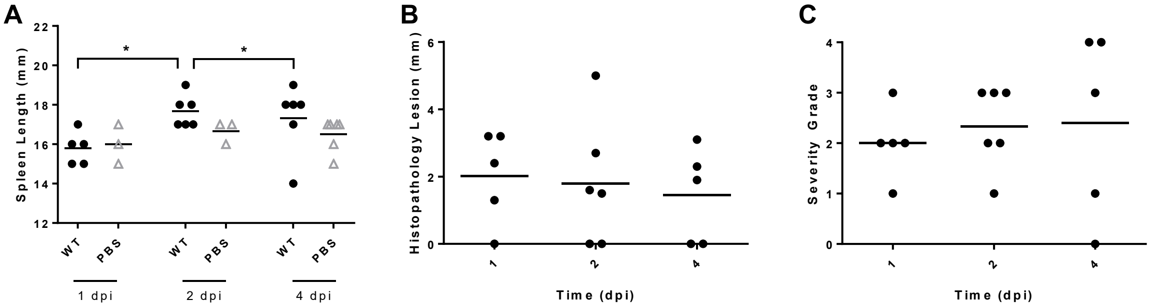

FIG S1 The skin pathology in wildtype-infected mice does not increase in size or severity over time. (A) Spleen length (mm) for WT-infected and control mice. (B) Histopathology lesion length (mm) and (C) histopathology severity grading for WT-infected mice. Each data point represents an individual mouse and mean scores (horizontal bars) were analysed by two-sample unpaired t-test, *p≤0.05. No differences in histopathology lesion length or severity grade were identified over time with large variation present between mice.

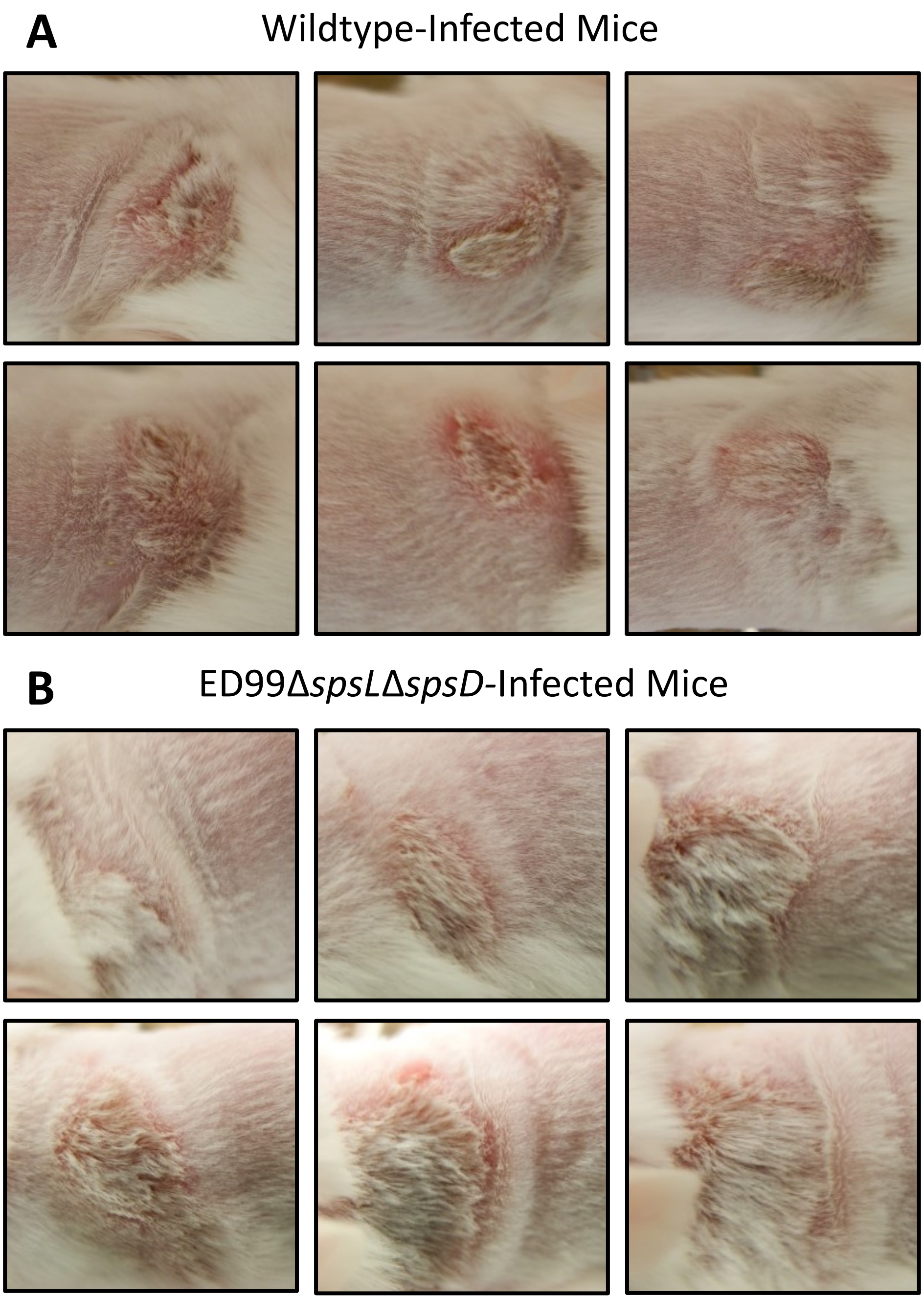

FIG S2 Gross examination identified larger, flatter surface lesions in ED99Δ*spsL*Δ*spsD*-infected mice. (A) WT-infected mice and (B) ED99Δ*spsL*Δ*spsD*-infected mice at 3 dpi. Each image represents a separate mouse on the day of euthanasia. Larger, flatter surface lesions are present in the ED99Δ*spsL*Δ*spsD*-infected mice compared to the WT-infected mice.

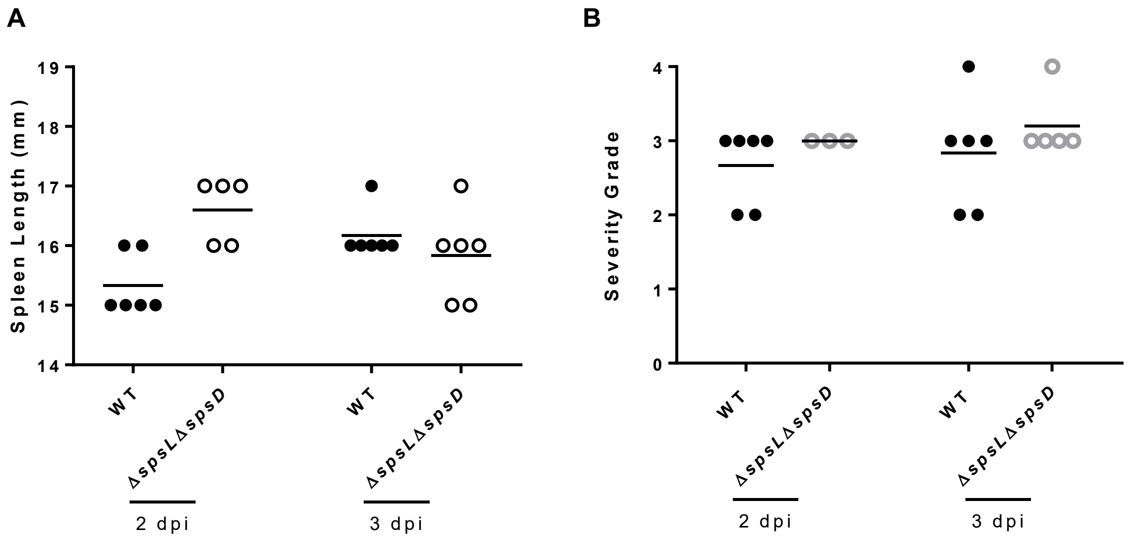

FIG S3 Deletion of *spsD* and *spsL* does not alter spleen size or the severity of infection. (A) Spleen length (mm) and (B) histopathology severity grades for WT- (filled circle), or ED99Δ*spsL*Δ*spsD*-infected mice (open circle). Each data point represents an individual mouse and mean scores are represented as horizontal bars. No differences in spleen size or severity grade were identified between the two experimental groups.

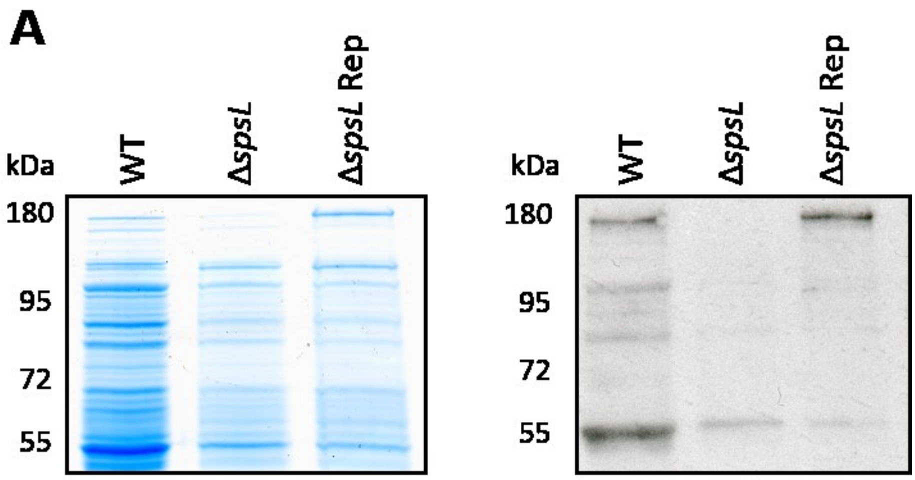

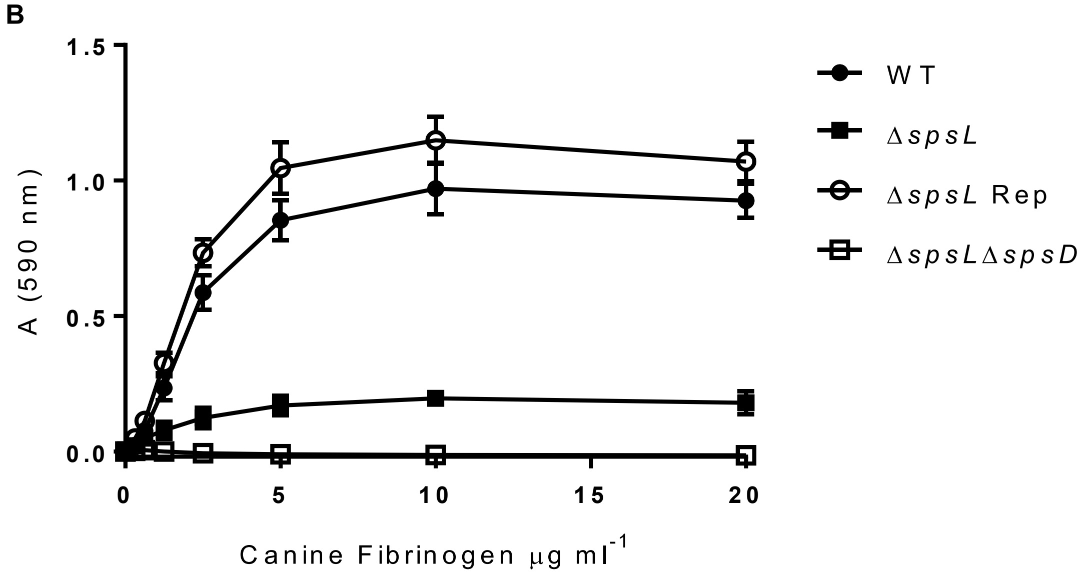

FIG S4 ED99Δ*spsL* Rep has Restored SpsL Expression and Canine Fibrinogen Binding. (A) Western blot using anti-SpsL N2N3 IgY antibody against cell wall-associated proteins of strains cultured to optical density (600 nm) of 0.6. (B) Bacterial adherence to canine fibrinogen of WT (closed circle), ED99Δ*spsL* (closed square), ED99Δ*spsL* Rep (open circle), and ED99Δ*spsL*Δ*spsD* (open square) strains. Each data point represents the mean value from three independent experiments; error bars represent SD.

TABLE S1 Primers used in this study to generate the Repaired strain ED99Δ*spsL* Rep

Primer	Sequence (5' to 3')	Function
spsL A	cctcactaaagggaacaaaagctgggtacCGGATGCAAATTTTCGAAT	pIMAY Construct
spsL B	ATGTTTTTCTTCTTCATTTTTGTACAC	pIMAY Construct
spsL C	gtgtacaaaaatgaagaagaaaaacatTCAATAAGAAAGTTATCTATA	pIMAY Construct
spsL D	cgactcactatagggcgaattggagctcTGTAACAGCAATACAACAAA	pIMAY Construct
MCS F	TACATGTCAAGAATAAACTGCCAAAGC	Confirm Construct
MCS R	AATACCTGTGACGGAAGATCACTTCG	Confirm Construct
OUT F	CATGCAACATAAGGCGACGAT	Confirm generation of
OUT R	TACGTCAAAGTGAACGTTTTGAC	Confirm generation of
Seq F	TTAATTTTCAAGGGTAGGAGTAGT	Sanger sequencing
Seq R	AAAGCCATCGTAAACCATAAAG	Sanger sequencing

Lower case sequences represent complementary sequences in allele replacement primers.
